# Implementation barriers and facilitators of Moyo foetal heart rate monitor during labour in public hospitals in Nepal

**DOI:** 10.1080/16549716.2024.2328894

**Published:** 2024-04-05

**Authors:** Ashish KC, Mikaela Rönnbäck, Urja Humgain, Omkar Basnet, Pratiksha Bhattarai, Anna Axelin

**Affiliations:** a School of Public Health and Community Medicine, Sahlgrenska Academy, University of Gothenburg, Gothenburg, Sweden; b Department of Women’s and Children’s Health, Uppsala University, Uppsala, Sweden; c Research Division, Golden Community, Lalitpur, Nepal; d Department of Nursing Science, University of Turku, Turku, Finland

**Keywords:** Moyo, fetal heart rate monitoring practice change, barriers for implementation, facilitators for implementation, Nepal

## Abstract

**Background:**

Globally, every year, approximately 1 million foetal deaths take place during the intrapartum period, fetal heart monitoring (FHRM) and timely intervention can reduce these deaths.

**Objective:**

This study evaluates the implementation barriers and facilitators of a device, Moyo for FHRM.

**Methods:**

The study adopted a qualitative study design in four hospitals in Nepal where Moyo was implemented for HRM. The study participants were labour room nurses and convenience sampling was used to select them. A total of 20 interviews were done to reach the data saturation. The interview transcripts were translated to English, and qualitative content analysis using deductive approach was applied.

**Results:**

Using the deductive approach, the data were organised into three categories i) changes in practice of FHRM, ii) barriers to implementing Moyo and iii) facilitators of implementing Moyo. Moyo improved adherence to intermittent FHRM as the device could handle higher caseloads compared to the previous devices. The implementation of Moyo was hindered by difficulty to organise training ondevice during non-working hours, technical issue of the device, nurse mistrust towards the device and previous experience of poor implementation to similar innovations. Facilitators for implementation included effective training on how to use Moyo, improvement in intrapartum foetal monitoring and improvement in staff morale, ease of using the device, Plan Do Study Act (PDSA) meetings to improve use of Moyo and supportive leadership.

**Conclusion:**

The change in FHRM practice suggests that the implementation of innovative solution such as Moyo was successful with adequate facilitation, supportive staff attitude and leadership.

## Paper Context

- **Main findings:** Before the Moyo implementation, foetal heart rate monitoring was sub-optimal in the hospitals, which changed after introduction of the device, as it helped early display of foetal heart rate in the monitor and supported communication with women during the labour and delivery.
- **Added knowledge:** Implementation of Moyo in low-resource setting requires an interdisciplinary approach with continuous support to health care providers on how to correctly read Moyo, maintenance of device and management of false reading.
- **Global health impact for policy and action:** The global efforts to accelerate reduce preventable intrapartum related neonatal death requires contextual understanding of clinical context for effective implementation of Moyo.

## Introduction

Each year, approximately, 1 million of the world’s stillbirth deaths occur during the intrapartum period [1]. Ninety-eight per cent of the global stillbirths occur in low-resource settings, and over 50% of these occur in South Asia, due to poor intrapartum care [2]. To reduce intrapartum deaths and improve intrapartum care, monitoring of the foetal wellbeing during intrapartum period and early detection of foetal distress is of paramount importance [3,4]. The early detection of intrapartum foetal distress through changes in foetal heart rate can facilitate timely decision of intervention to avoid intrapartum stillbirth and intrapartum related neonatal mortality [3].

While complex and continuous Foetal Heart Rate Monitoring (FHRM) devices are widely used in high-resource settings, low-cost and easy-to-use devices will be suitable in low-resource settings [5]. A systematic review on strategies to improve FHRM in low- and middle-income settings suggested use of FHRM devices that are simple, affordable, robust, safe, reliable and sustainable to use [6]. Among the different affordable devices for FHRM, Moyo is one of them [7].

Moyo is a novel, low-cost FHRM device developed in 2015 for intrapartum foetal heart rate monitoring in low-resource settings. It is an electronic and portable device designed for both continuous and intermittent use in FHRM. Moyo quickly detects foetal heart rate over a wide range and can distinguish between foetal and maternal heart rates and is easy and reliable to use [8–10]. It records foetal heart rate, displaying the last 30 minutes of recording on a visual screen, and has an audio-visual alarm for irregular foetal heart rate. Moyo is designed to assist health care providers to correctly manage foetal conditions and thus perform interventions in a timely manner [11]. A study in Tanzania reported that before the introduction of Moyo, health care providers felt FHRM overwhelming [12]. After the implementation of Moyo in the hospital of Tanzania, health care provider still lacked clarity on when to use Moyo and differentiate maternal heart rate with foetal heart rate [8–10], indicating a further need to understand the facilitators and barriers to implement Moyo in low- and middle-income countries.

In Nepal, FHRM devices such as stethoscope, fetoscope and handheld Dopplers were used for intermittent intrapartum monitoring [13]. Despite an unprecedented increase in institutional childbirth between 2000 and 2017, the country faced poor adherence to WHO guideline of FHRM every 15–30 minutes [14]. As a result, increased number of stillbirths were observed when there was decrease in adherence to intrapartum monitoring [15]. To improve health care and reduce intrapartum-related mortality, the ‘Scaling Up Safer Birth Bundle Through Quality Improvement in Nepal (SUSTAIN)’ quality improvement package was implemented in eight public hospitals in Nepal. One of the innovations included in the quality improvement package was Moyo, a device for FHRM to improve the quality of intrapartum care [16]. This study aims to explore the implementation barriers and facilitators for the use of Moyo as FHRM during labour.

## Methods

The study followed a qualitative descriptive study design to evaluate the implementation of Moyo for FHRM. The qualitative study was implemented between October 2020 and May 2021 in the first four hospitals of the SUSTAIN study in Nepal.

## Study settings

The SUSTAIN study had eight public hospitals where Moyo and neonatal resuscitation package was implemented between May 2019 and April 2021. The quality improvement package in these hospitals was implemented in a stepped wedge pattern, with interventions introduced in each hospital at different time points. Four hospitals were purposively selected using maximum variation sampling. To obtain sufficient variation, two hospitals where implementation of the SUSTAIN project had not been very successful and two hospitals where implementation had been successful were invited to participate. The four hospitals were Surkhet Provincial, Bheri hospital, Seti Provincial and Janakpur hospital. Surkhet hospital was a provincial referral hospital in Karnali province which provided comprehensive and emergency obstetric care services with annual 3,847 women delivering in 2019. The hospital had 10 nurses trained in midwifery skills to provide intrapartum care. Bheri hospital was a provincial referral hospital in Lumbini province which provided Comprehensive Emergency and Obstetric Care (CEOC) services with 4,132 women delivering in 2019. The hospital had 12 nurses trained to provide intrapartum care. Seti Provincial hospital was a provincial hospital in Sudurpachim Province which provides CEOC services with 6,277 women delivering in 2019. The hospital had 15 nurses trained to provide intrapartum care. Janakpur provincial hospital was a CEOC referral center with 14,300 women delivering in 2019 with 12 nurses to provide the care.

### Implementation strategy of Moyo for FHRM

There were four strategies to the implementation of Moyo as part of SUSTAIN package, *first*, strengthening hospital leadership to set up a system to benchmark for foetal heart rate monitoring and continuously review of progress to overcome the bottlenecks in the system; *second*, empower and capacitate nursing staff on intrapartum foetal heart rate monitoring using Moyo; *third*, facilitate through Plan-Do-Study-Act cycle to improve the coverage of intrapartum and *finally*, continuously monitor the use of Moyo.

### Participant selection

In line with convenience sampling, available nurses from four hospitals were invited to participate as per following inclusion criteria 1) worked in the labour and delivery room to provide intrapartum care, 2) had received training on Moyo, and 3) participated in the weekly meeting to discuss the problems on intrapartum monitoring and plan on how to improve care and implement the change using the Plan-Do-Study-Act cycle. All the participants were females with a mean age of 34 years and all of them had at least 4 years of practice in their profession (Table 1).

**Table 1.** Participant table from the hospitals.

Profession	Education	Age (yrs.)	Years of practice	Years working in current ward
Nursing Officer	MSc Nursing	34	10	4
Hospital Nursing Inspector	BSN, M. in Sociology	42	22	10
General Nursing	BSN	39	20	2
Staff Nurse	BSN	32	12	5
Staff Nurse	SANM	40	14	1,5
Staff Nurse	PCL Nursing	33	20	3
Staff Nurse	BSN	30	8	3
Staff Nurse	BSN	31	11	1
Staff Nurse	PCL Nursing	25	7	4
Staff Nurse	BSN	29	12	2
Staff Nurse	MSc Nursing	24	4	2,5
Staff Nurse	ANM	52	27	10
Staff Nurse	ANM	26	8	1,5
Staff Nurse	ANM	43	22	8
Staff Nurse	ANM	33	12	11
Staff Nurse	BSN	33	10	1
Staff Nurse	BSN/PCL Nursing	33	11	3
Staff Nurse	BSN	46	28	23
Staff Nurse	PCL Nursing	24	4	1
Staff Nurse	BSN, PCL Nursing	35	12	1

### Data collection

A semi-structured interview guide was developed on the basis of i-PARIHS framework. The framework guided the exploration of the factors influencing on implementation outcomes [17,18]. Two pilot interviews were conducted before finalising the interview guide, analysis of which were not included in the study. The final interview guide consisted of nine open questions to explore the facilitators and barriers present in the implementation of Moyo FHRM (Appendix A).

The data collectors who conducted the interviews were Nepalese researchers who were not involved in the implementation of the SUSTAIN project and who had experience in qualitative research. They were trained by the lead researcher (AA) to conduct qualitative interviews with reflexivity and to use bracketing to suspend their assumptions and previous experiences from the study process. Eligible participants were approached via email and data collection was done via video calls on Zoom. The interview was taken in Nepali language and recording of the interview was done with the consent from the participants. A total of 20 participants were interviewed from the four hospitals and analysis was done after each two interviews to check for data saturation [19]. Data saturation was the basis to conclude the sample size and it was achieved when no additional substantive information was obtained after 16 interviews. The interviews were then transcribed and translated into English for further analysis within the international research team.

### Data analysis

The analysis process followed qualitative content analysis with the deductive approach described by Elo and Kyngäs [19]. Practice change, barriers and facilitators for successful implementation were used to organise the data deductively. The interview transcripts were read carefully, and one researcher (MR) coded the data. After the codes were grouped under the three categories of the deductive framework, subcategories were developed following the principles of inductive content analysis to generate the findings under each of the three categories. The lead researcher AA closely supervised MR throughout the analysis. The supervisor also became familiar with the interview transcripts. Any disagreements in the analysis were resolved through discussion. Throughout the analysis process, findings were regularly discussed with the Nepalese researchers to maintain credibility.

The participants had full knowledge about the interview and data collection processes. They were informed that the interviews will be recorded, anonymized, and handled with confidentiality. The study was approved by the Ethical Review Board of Nepal Health Research Council (number-110/2019). Informed consent was received both via email and verbally which was recorded before the interview.

## Results

Using the deductive approach, the data were organised in three categories i) change in practice of FHRM, ii) barriers to implementing Moyo and iii) facilitators of implementing Moyo. The subcategories under each category were developed following the principles of inductive content analysis (Table 2).

**Table 2.** Three main categories and their sub-categories.*

Sub-categories	Main categories
Better monitoring for more patients	Practice change-Improved intrapartum FHRM
Ability to provide continuous remote monitoring	
Problems in design and technology	Barriers for the successful implementation of FHRM with Moyo
Negligence and mistrust towards Moyo	
Lack of human resources	
Previous failure to implement new innovations	
Covid-19 helped and hindered	
Motivating and well-executed training on Moyo	Facilitators for the successful implementation of FHRM with Moyo
Improved intrapartum care and staff morale	
Moyo facilitated easy FHRM and multi-tasking	
PDSA meeting facilitated improved practice	
Leadership supporting practice change	
Covid-19 helped and hindered	

*Using deductive approach main category was developed and subcategories under each category were developed using principles of inductive content analysis.

### Practice change - improved intrapartum FHRM

Since the implementation of SUSTAIN, most nurses reported that their practice of FHRM had improved significantly. SUSTAIN brought many new changes, both with the Moyo device itself and with the help and support of the external facilitators. The change in practice following the introduction of Moyo was described by nursing staffs in two subcategories: better monitoring for more patients and the ability to provide continuous remote monitoring.

#### Better monitoring for more patients

With the help of Moyo and the SUSTAIN programme, the hospitals were able to increase the number of patients receiving regular monitoring. Nurses reported that all hospitals were using the new Moyo device in their practice. Prior to the introduction of Moyo, staff were unable to provide high-quality monitoring in all cases because the old equipment – stethoscope, fetoscope and Doppler – required more resources and lacked adequate monitoring qualities. The old equipment was particularly difficult for inexperienced staff to use, sometimes resulting in unreliable monitoring.

Due to inadequate number of staffs, it was very difficult to perform intrapartum FHR monitoring but after we received “Moyos”, we have been able to practice intrapartum monitoring regularly in our hospital. Interview 01

#### Ability to provide continuous remote monitoring

Because the Moyo could be used for continuous and remote monitoring, nurses were able to perform systematic automated FHRM during labour. This was a major improvement over the old intermittent monitoring devices that required frequent bedside monitoring. The old devices made frequent FHRM difficult under high caseload conditions and were risky for the nursing staff during Covid-19, as they often had to be close to the patients. A nurse at Hospital 4 explained-

For using doppler, we have to regularly visit the patient, touch them and use gel every half an hour but with Moyo we can have a continuous monitoring from a distance. Interview 05

### Barriers for the successful implementation of FHRM with Moyo

Several barriers were discussed by the hospital nursing staffs. They were related to technical problems, nurses’ negative attitudes and mistrust of the device, organisation of training, and previous failures in implementing new innovations.

#### Problems in design and technology

The most common barrier seemed to be that Moyo sometimes displayed an incorrect FHR. The nurses also identified other technical problems, such as short battery life. In busy hospitals with high caseloads, such as those in Nepal, it was problematic when the battery run out and nurses had to find time to recharge the devices. Staff also discussed that the alarm sound on Moyo was sometimes lost or not heard at all when the FHR fluctuated. It was also mentioned that the belt used to attach Moyo to the patient was too long and became contaminated from contact with the floor. It was further explained that the belt needs to fit correctly, not too tight and not too loose, for the FHR to be displayed correctly. These were some of the barriers presented, here explained by a nurse in Hospital 1:

Sometimes the battery doesn’t work well. Even after charging Moyo to full and apply then after a while battery is also gone. It shut self-down sometimes, it is happening so … we have 3 Moyo and 2 doesn’t work well. Interview 09

#### Negligence and mistrust towards Moyo

Nurses mentioned that some nursing staffs were irresponsible in their work, not using or cleaning equipment properly. Another major problem seemed to be nursing staff negligence. Negligence was based both on high caseload situations where stress made nurses reluctant to use new equipment, but also on mistrust of Moyo. Mistrust seemed to be based on the most recurring barrier, that the Moyo gave false readings and was therefore considered unreliable. These erroneous readings were considered to be false FHRs in stillbirths and false abnormal FHRs in normal cases, as well as unclear FHRs. Although false readings appeared to be rare, as nurses explained that Moyo worked well in most cases, these occasional false readings caused confusion and doubt among staff. The aspect of Moyo’s unreliability discouraged some nurses from using the device. Instead, they used other FHRM devices or methods to confirm a questionable Moyo reading, or in some cases, nurses used the other device exclusively for FHRM, rejecting Moyo altogether. Here is an explanation of how other methods were used when Moyo showed incorrect readings and confusion in Hospital 2:

Sometime back it was confusing due to aortic sound of the mother in one or two cases like in stillbirth case, FHS was shown in MOYO after fetal death, which confused us, and we have reported that as well. So, for confirmation we took maternal pulses. Interview 08

#### Lack of human resources

In hospitals 1 and 3, nurses faced some problems during the training. Staff shortages prevented them from taking time for training. Therefore, they had to attend the training during the day and then immediately work shifts or go straight from night duty to the training. *This busy schedule left the nurses exhausted and unable to perform well in the training. In Hospital 1, a nurse also described the training as insufficient and not given to all professions involved in patient care*:

During that time, they explained little, in short on the equipment … If there was detailed information on the equipment, then it would have been better … Until now, all is fine about the training but not only being focused on nurses if doctors were also included, it would have been better. Interview 09

#### Previous failure to implement new innovations

A nurse from Hospital 1 explained how staffs were initially sceptical about SUSTAIN and the innovations because the hospital had a history of failing to implement new interventions. She said she doubted that Moyo would be helpful or even used in practice. Previously, they implemented new innovations only during training period, but had been unsuccessful in implementing them in clinical practice. The negative experiences of the past seemed to influence the staff’s views:

We were puzzled if these interventions would come into action at our hospital and whether we would be able to make use of all these interventions. As per our old experiences, many techniques were only limited in the training program and did not come into play thereafter … So, learning was limited only during the training period. Interview 04

### Facilitators for the successful implementation of FHRM with Moyo

Several facilitators were discussed by the hospital staffs. These included good training on how to use the Moyo, improved intrapartum care and staff morale, ease of use of the device, PDSA meetings, and supportive leadership. In addition, the COVID-10 pandemic played a role in the implementation.

#### Motivating and well-executed training on Moyo

External facilitators conducted training programmes at the beginning of implementation to teach staff about Moyo and PDSA. Most nurses were satisfied with the training, both the content and the expert facilitators who delivered it. They found the training to be understandable, relevant, and useful. The whole programme was conducted in a positive environment and was staff oriented. *Nurses described how the training refreshed their knowledge, supported their practice, and used the skills taught in their daily work on the wards. Staff also explained how knowledge facilitated practice and how successful training motivated further training*. The training also seemed to motivate the use of Moyo on the wards and improve overall practice. A nurse at Hospital 2 said:

We already have the knowledge but after the training the information is updated and also, we gain new knowledge. The trainers motivate us to use the equipment and after training we also feel motivated, and we do the work as well. The training was adequate, and we are following it until now as well. Interview 07

#### Improved intrapartum care and staff morale

Nurses expressed that Moyo improved their daily routine practice and helped to improve intrapartum care. Nurses explained that most women were cooperative about using Moyo. Once the device was explained to them and they understood the benefits of the practice, they were positive about wearing it. As the fetal heart rate was displayed on the device itself, women were able to observe their own foetuses and became more involved in their care. Patients asked questions and showed interest, and some reported to staff when they witnessed an abnormal fetal heart rate, which was helpful in practice. Moyo seemed to help with communication between women and midwives. This was discussed in hospital 3, for example:

Patients and visitors themselves tell us that the color is changing and ask about the reason for the change. Moyo has greatly improved communication between midwife and patient… No patient has been unsupportive so far. Interview 05

In addition to improved communication with women, the constant pursuit of new knowledge and the belief that learning is never enough to motivated staff to participate in Moyo’s training and work. Nurses mentioned that adequate knowledge facilitated their work. In addition, staffs in most hospitals explained that they had sustained teamwork where nurses, but also doctors and other staffs, coordinated interprofessional care and supported each other in providing good care to patients. This was explained in hospital 1:

I have worked in various hospitals previously but when I compare the work we are currently doing; I feel that intrapartum monitoring is very effectively done. Not praising our own work, but we work with such dedication … I think, very few government hospitals have such sincerity while providing nursing care. Interview 04

#### Moyo facilitated easy FHRM and multi-tasking

Nurses described how Moyo had alarm and recording functions and was able to monitor both foetal and maternal heart rates. The alarm functions assist nurses by indicating when foetal heart rate is normal or abnormal. Hospital staff explained how Moyo enabled them to measure foetal heart rate remotely, and to record the FHR. Staff did not have to attend to the mothers all the time after placing the Moyo but were still able to monitor the foetal heart rate continuously from a distance, making FHRM possible with limited staff and allowing them to multi-task by performing other tasks at the same time as monitoring. A staff member from Hospital 3 explained as

… I am happy to use it because monitoring can be done and at the same time, other activities can be performed. If the fetal heart rate becomes irregular, it immediately goes to the red-zone … if the heartrate goes up or down, the indicator gives us signal about some situation which makes easier for us to take action. Interview 06

#### PDSA meeting facilitated improved practice

In PDSA meetings, nurses from all hospitals explained how work performance was reviewed and analysed through frequent data collection from the hospital environment. Problems were discussed and solutions provided. Issues were presented and practical changes were implemented in the hospitals. PDSA meetings helped improve staff behaviour and practice and provided strategies for effective care, as described here in Hospital 3:

PDSAs have been effective as it has helpful in improving recording and reporting as well … PDSAs have helped to solve common gaps and problems, change our behaviour and improve our clinical practice … These strategies have been effective to provide care to the mothers and newborns. Interview 03

#### Leadership supporting practice change

Good leadership was described as a facilitator as hospital managers supported Moyo use and trusted, nurses’ abilities rather than questioning their work. Appropriate inter-professional communication and trust in the use of Moyo by other professions seemed to support the use of Moyo as an FHRM practice. Nurses explained that managers and doctors visited the wards, mobilised resources well, and gave feedback on what was done well and what could be improved, supervising and guiding practice. This was mentioned in hospital 3:

Yes, we are getting more support than before on our work … . In-charge of the labour room also monitors all activities and makes sure that if the data is recorded or not, Moyo is used or not … Head of Department also comes twice a day to the labor room. Interview 05

### Covid-19 helped and hindered

Finally, the focus was on the then ongoing COVID-19 pandemic, which affected the practice and implementation of Moyo both positively and negatively, although it seemed to hinder the most. Nurses expressed the client caseload decreased during COVID-19 pandemic, which meant that hospitals had lesser deliveries than before, making work easier for staff. They also mentioned that the pandemic did not affect the implementation of Moyo or worsen intrapartum care at most sites. At the same time, staff were afraid of becoming infected, which made them reluctant to be around patients and monitor them, putting themselves at risk. As mentioned above, Moyo helped in practice with its continuous quality monitoring, but staff were careful of virus transmission, and shortage of nursing staff due to the COVD-19 infection. Finally, COVID-19 pandemic reduced the number of the PDSA meetings that were planned in the hospitals. This is described in Hospital 4 :

No, COVID did not affect implementation of SUSTAIN project. It didn’t make a difference, but fetal heart rate monitoring was not done as required. Interview 11

## Discussion

The study explored facilitators and barriers for implementation of Moyo to bring change in the FHRM practice in the hospitals. The inadequate FHRM practice before Moyo implementation improved after introduction of the device as it helped early detection of foetal heart rate and supported communication with women during the labor and delivery. Nevertheless, there was confusion between maternal heart rate with foetal heart rate displayed by Moyo and the need to frequently maintain the device were the implementation barriers. The training and motivation by facilitators on rationale and how to use Moyo through training and regular weekly PDSA meeting, the display of colour code in foetal heart rate in Moyo helped improve early communication between nurse and women were facilitators for implementation of Moyo.

The findings are in line with previous Moyo studies conducted in similar low-resource settings. The Improvement in FHRM, where Moyo managed to monitor higher caseloads because of its easy to use and display monitoring feature, was also seen in previously conducted studies in low resource settings in Africa [10]. There were similarities with other studies to support how Moyo facilitated FHRM by detecting higher proportion of abnormal foetal heart rate than other intermittent devices [11,20]. Feature of early detection of foetal heart rate by Moyo with better sensitivity to detect abnormal FHR [21] mirrored the experiences of nurses from this study. The new and interestingly finding was the improved communication between nurses and women as Moyo displayed foetal heart rate and alarmed if the foetal heart rate was abnormal. The visible foetal heart rate in the device made the women feel that nurses committed to the foetal’s wellbeing during labour. Co-design of medical devices and active involvement of pregnant women in implementation efforts (design thinking) is a promising way to improve peripartum care in low- and middle-income countries [22].

The reliability of new medical device readings is an absolute necessity for its clinical use. The Moyo is a reliable medical device [23], but in this study the false readings caused nurses to distrust the device. The same was found in a study in Tanzania, which showed that midwives were more likely to use FHRM devices if they provided reliable fetal heart rate readings [24]. Sufficient knowledge of the device and its source of measurement error seemed to be very important for successful intrapartum monitoring, and this was also discussed by nurses in this study [10,20]. Training and especially PDSA meetings could be an important opportunity to understand and manage these reliability issues so that they do not pose a threat to successful implementation.

With reliable equipment, it is easier to overcome staff’s negative attitudes towards the new equipment, which can be partly explained by overwhelming workload [25]. When workloads are high, it is difficult to find resources to implement new practices because clinical routine work seamlessly when the old routine practices are followed. According to the normalizing process theory, any new intervention becomes an integral part of routine practice when it is normalised, e.g. when it is no longer overwhelming to the nurses and staffs [26]. This normalisation is developed in a multi-professional collaboration that requires commitment and collective action, which is shown in our study. The dependence of staff on the research team and other contextual factors was further supported by findings from a study in Nigeria, Africa, where failure to adapt to FHRM protocols appeared to be dependent on contextual factors such as lack of leadership support, insufficient staff or equipment, and high workload [27].

This study used the i-PARHIS framework as a theoretical basis, which helped to provide a comprehensive understanding of the barriers and facilitators to FHRM implementation [18]. In addition, pilot interviews helped us to revise and develop a robust interview guide. The study maintains credibility by selecting participants from both successful and unsuccessful sites, in order to collect broad perspectives and rich data. Data analysis, carried out in parallel with data collection to ensure data saturation, also supported the credibility of our findings.

## Limitation

The study does have some limitations. The researchers who conducted the interviews were already involved in the study design of SUSTAIN, which may explain the over-representation of positive outcomes. Participants may therefore have been reluctant to share negative views or may not have participated if they had more negative than positive views. As the interviews were translated from Nepali to English, some information may have been lost in translation. Our findings can be transferred to other low- and middle-income contexts with some caution e.g. in Tanzania, where scale up of Safer birth bundle in health facilities ongoing to improve adherence of Moyo for FHRM.

## Conclusions

The findings suggest that there have been changes in practice with improved intrapartum FHRM following the implementation of Moyo in these hospitals with an interdisciplinary approach. In these low-resourced settings, there is a need for continuous support to health care providers on how to correctly read Moyo, maintenance of device and management of false reading.

The global efforts to accelerate reduction in neonatal mortality require effective implementation of innovative and low-cost interventions such as Moyo. In the light of Sustained Development Goals (SDGs), which place greater emphasis on sustainable approaches to care, the transfer of skills to health workers is critical, requiring appropriate facilitation, management and continuous monitoring of contextual factors.

## Data Availability

The datasets generated and/or analysed during the current study cannot be made publicly available due to the involvement of information of individuals disclosing identifiers.

## Acronyms

ANM: Auxiliary Nurse Midwife
BSN: Bachelor of Science in Nursing
FHR: Fetal Heart Rate
FHRM: Fetal Heart Rate Monitor
i-PARIHS: Integrated Promoting Action on Research Implementation in Health Services
MSc: Master of Science in Nursing
PCL: Proficiency Certificate Level
PDSA: Plan-Do-Study-Act
SDGs: Sustainable Development Goals
SUSTAIN: Scaling Up Safer Birth Bundle Through Quality Improvement in Nepal
SWOT: Strengths Weaknesses Opportunities Threats

## Appendix A. Interview guide

Semi structured interview guide for individual interviews

Gender and profession of the interviewer:

Introduction questions:

● Gender:

● Age:

● Profession and education:

● Years of practice:

● Hospital and ward:

(Participants must have been working at the ward during the whole SUSTAIN study process.)

If needed, please use under each nine main questions relevant probes, such as please tell me more about that, could you explain that in more detail, that is interesting, what did you do, what were you thinking about it, how did it make you feel, did you have any other options, have you had similar experiences before. The most important thing is to give enough space and time for a person to think and tell about experiences.

(1) What do you know about the SUSTAIN project? Can you briefly explain it?

(2) Describe your current practices in Intrapartum monitoring.

(3) What new has the SUSTAIN bundle brought to your practice?

(4) What has been your experiences on using Moyo fetal heart rate (FHR) monitors during intrapartum care? (Innovation)
Follow up questions: Enablers to why it worked/barriers to why it didn’t work for FHR monitoring? Your own thoughts on the importance of using Moyo FHR monitors? (Recipients; motivation, goals, skills etc.)

(5) How did you experience the 2-month training period on the methods of the SUSTAIN project? How did you experience the Moyo FHR monitoring trainings,? (Facilitation)

(6) *Follow-up questions: Were there differences on how the trainings were successful or not and WHY between 1), 2) and 3)? In what form did you get feedback? How is this feedback going to help you in your work in intrapartum care/newborn resuscitation in clinical practice? What was helpful? What wasn’t? What could’ve been done differently to facilitate trainings and hence the implementation?*

(7) How did you experience the 1) Facilitators from the Golden community AND your own hospital, 2) PDSA-meetings? (Facilitation)

(8) *Follow-up questions: Did these strategies help to implement SUSTAIN into your practice, why or why not? Was there a difference in implementing intrapartum (Moyo FHR monitors), if so why? Enablers why implementation worked/Barriers why it didn’t work fully? What could’ve been done differently to facilitate implementation?*

(10) How did you experience 1) the Leadership; have the leaders visited the unit/ward? Have you discussed about SUSTAIN with the leaders? and 2) Other Resources during the SUSTAIN project; have you received some extra resources from the SUSTAIN project to your work? (context)
Follow up questions: Thoughts on support and knowledge to make implementation possible? Enough of it? What barriers were there? Enablers? How was the teamwork? Have you received any feedback from the leadership during the process? Has the corona epidemic impacted on the implementation?

Thank you for your time and help for the development of care!
